# Transmission dynamics of the 2022 mpox epidemic in New York City

**DOI:** 10.1038/s41591-025-03526-9

**Published:** 2025-03-25

**Authors:** Jonathan E. Pekar, Yu Wang, Jade C. Wang, Yucai Shao, Faten Taki, Lisa A. Forgione, Helly Amin, Tyler Clabby, Kimberly Johnson, Lucia V. Torian, Sarah L. Braunstein, Preeti Pathela, Enoma Omoregie, Scott Hughes, Marc A. Suchard, Tetyana I. Vasylyeva, Philippe Lemey, Joel O. Wertheim

**Affiliations:** 1 Institute of Ecology and Evolution, University of Edinburgh, Edinburgh, UK; 2 Bioinformatics and Systems Biology Graduate Program, University of California San Diego, La Jolla, CA USA; 3 Department of Biomedical Informatics, University of California San Diego, La Jolla, CA USA; 4 Department of Medicine, University of California San Diego, La Jolla, CA USA; 5 Department of Computer Science and Engineering, University of California San Diego, La Jolla, CA USA; 6 New York City Department of Health and Mental Hygiene, Public Health Laboratory, New York, NY USA; 7 Department of Biostatistics, Fielding School of Public Health, University of California Los Angeles, Los Angeles, CA USA; 8 New York City Department of Health and Mental Hygiene, Bureau of Hepatitis, HIV and Sexually Transmitted Infections, Long Island City, NY USA; 9 Department of Biomathematics, David Geffen School of Medicine, University of California Los Angeles, Los Angeles, CA USA; 10 Department of Human Genetics, David Geffen School of Medicine, University of California Los Angeles, Los Angeles, CA USA; 11 Department of Population Health and Disease Prevention, Joe C. Wen School of Public Health, University of California Irvine, Irvine, CA USA; 12 Department of Microbiology, Immunology and Transplantation, Rega Institute, Laboratory for Clinical and Epidemiological Virology, KU Leuven, Leuven, Belgium

**Keywords:** Phylogenetics, Epidemiology

## Abstract

The 2022 global mpox epidemic was caused by transmission of MPXV clade IIb, lineage B.1 through sexual contact networks, with New York City (NYC) experiencing the first and largest outbreak in the United States. By performing phylogeographic analysis of MPXV genomes sampled from 757 individuals in NYC between April 2022 and April 2023, and 3,287 MPXV genomes sampled around the world, we identify over 200 introductions of MPXV into NYC with at least 84 leading to onward transmission. These infections primarily occurred among men who have sex with men, transgender women and nonbinary individuals. Through a comparative analysis with HIV in NYC, we find that both MPXV and HIV genomic cluster sizes are best fit by scale-free distributions, and that people in MPXV clusters are more likely to have previously received an HIV diagnosis and be a member of a recently growing HIV transmission cluster. We model MPXV transmission through sexual contact networks and show that highly connected individuals would be disproportionately infected at the start of an epidemic, which would likely result in the exhaustion of the most densely connected parts of the network, and, therefore, explain the rapid expansion and decline of the NYC outbreak. By coupling the genomic epidemiology of MPXV and HIV with epidemic modeling, we demonstrate that the transmission dynamics of MPXV in NYC can be understood by general principles of sexually transmitted pathogens.

## Abstract (web summary)

Analysis of genomic and epidemiology data from 2022 in New York City show similarities between the dynamics of MPXV and HIV transmission, and highlight the role of heavy-tailed sexual contact networks in disease transmission.

## Main

In May 2022 a global mpox epidemic was identified, starting with the diagnosis of a case in the United Kingdom, which was swiftly followed by localized outbreaks across the world. International transmissions of mpox virus (MPXV), particularly clade IIb, lineage B.1, seeded these outbreaks, and these transmission events were distinguishable due to an elevated evolutionary rate attributable to APOBEC3-associated hypermutation^1–6^. By the end of 2022 the global case count of this epidemic exceeded 80,000 and the United States was the country with the largest number of cases, at 30,092 (ref. ^7^), with the earliest importations into the United States likely to be coming from Western Europe^4^. In the United States, New York City (NYC) experienced the first and largest major outbreak, with 3,821 cases diagnosed by the end of 2022 (refs. ^8,9^). NYC also conducted the first mpox vaccination campaigns in the country, initiated in June 2022 (ref. ^10^).

During the 2022 epidemic, MPXV is believed to have primarily spread through sexual contact networks among men who have sex with men (MSM)^11,12^, whereas previous outbreaks were the result of zoonotic, household and, in rare cases, hospital-based transmissions^13^. These outbreaks were typically self-contained and with limited human-to-human transmission. Epidemic modeling suggests that the rapid global emergence, expansion and decline of mpox during the 2022 epidemic could be explained by the transmission of MPXV through heavy-tailed sexual contact networks^14–17^, a heterogeneous network in which a subset of individuals have a disproportionately high number of contacts. Such networks account for the transmission patterns of other sexually transmitted pathogens, including human immunodeficiency virus type 1 (HIV-1)^18–21^, hepatitis C virus^22^ and *Treponema pallidum pallidum* (the etiological agent of syphilis)^23–25^. Phylodynamic analyses of sexually transmitted pathogens, including HIV, have shown that they recapitulate these heavy-tailed sexual contact networks^19–22^; however, these hypothesized dynamics have not yet been confirmed for MPXV by empirical analyses.

The mpox outbreak in NYC peaked shortly after the initiation of its vaccination campaign, in which two doses were administered 1 month apart, but it is unclear whether the subsequent declining incidence of mpox was due to vaccination, behavioral changes, sexual contact network dynamics, or some combination thereof^26^. Long-term public health surveillance in NYC provides a distinct opportunity to examine whether the transmission dynamics of MPXV are consistent with those of other sexually transmitted infections, specifically HIV-1, for which molecular surveillance data have been collected for two decades and sexual transmission dynamics are well understood^18–21,27,28^. Here, we used phylogeographic and epidemiological modeling approaches to better understand the factors governing the trajectory of the mpox outbreak in NYC in the context of the global epidemic and the intersection between the MPXV and HIV transmission networks.

## Results

### Emergence of mpox in NYC

**Fig. 1:**
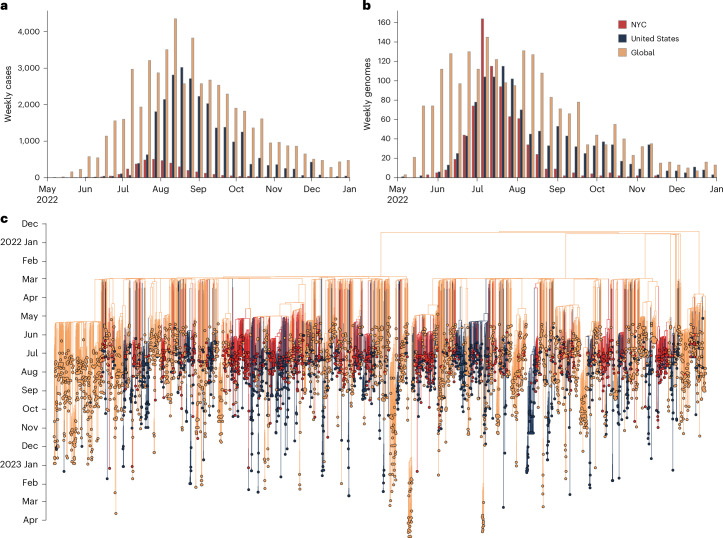
Emergence of the 2022 mpox epidemic in NYC. **a**, The number of cases of mpox per week in NYC, the United States and globally, where ‘United States’ refers to all of the United States excluding NYC and ‘global’ refers to all countries other than the United States. **b**, The number of sequenced genomes per week in NYC, the United States and globally. **c**, Time-calibrated phylogenetic tree of MPXV. Genomes are represented by circles at the tips of the phylogenetic tree, with tips and branches colored by location.

The mpox outbreak in NYC rapidly grew in the summer of 2022 (June–July; Fig. 1a), while the rest of the United States did not experience a marked increase in cases until the end of July. Mpox cases in NYC peaked in late July, approximately 1 month before the peaks in the rest of the United States and globally. A substantially higher percentage of diagnosed mpox infections were sequenced in NYC through to the end of April 2023 (19.7%; *n* = 760) than in either the rest of the United States (4.6%; *n* = 1,221) or the rest of the world (4.1%, *n* = 2,338), enabling a fine-grained phylogeographic analysis of the MPXV outbreak in NYC. More than 99% of the MPXV genomes from NYC were produced by the NYC Department of Health and Mental Hygiene (DOHMH) Public Health Laboratory (PHL).

**Fig. 2:**
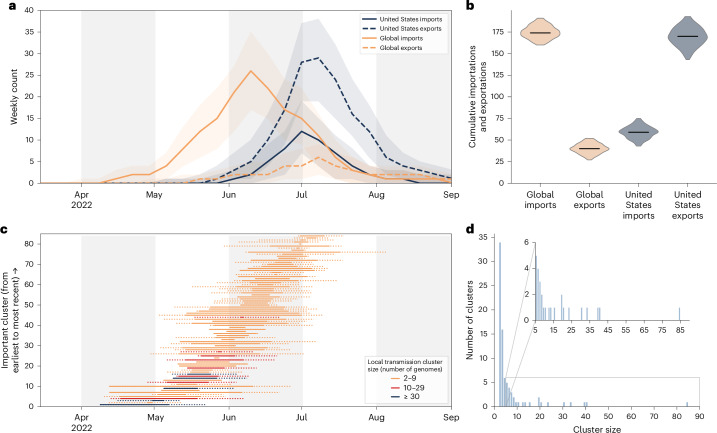
Importations and exportations of MPXV in NYC. **a**, The estimated number of MPXV importations into, and exportations out of, NYC per week. The center line indicates the median, and the shaded areas show the 95% HPDs of the estimates. **b**, Cumulative number of MPXV importations into and exportations out of NYC. The violins represent the 95% HPDs and the black bars indicate the medians. **c**, Estimated importation times for the 84 inferred MPXV NYC transmission clusters. The left limit of the solid horizontal line is the median time of the parent node of the MRCA of each transmission cluster, and the right limit is the median time of the MRCA (tMRCA) of the transmission cluster. The left limit of the dotted line is the lower bound of the 95% HPD of the time of the parent node, and the right limit is the upper bound of the 95% HPD of the tMRCA. **d**, Distribution of MPXV transmission cluster sizes in NYC.

To characterize the introductions and emergence of MPXV in NYC, we conducted a Bayesian phylogeographic analysis of MPXV sampled from April 2022 to the end of April 2023, with 757 high-quality genomes from NYC (756 sequenced by the NYC PHL), 1,127 from the rest of the United States, and 2,160 from the rest of the world^29^. We found that MPXV was likely to be cryptically circulating in NYC and the rest of the United States for 1–2 months before the earliest cases of the 2022 mpox outbreak were identified on 19 May and 17 May in NYC and the broader United States, respectively (Fig. 1c), in line with previous results^4^. We inferred that global importations of MPXV into NYC started in April, with a peak in mid-June (Fig. 2a), and identified a median of 174 global importations into NYC in total (95% highest posterior density [HPD], 160–191). Based on the available sampling, there were substantially fewer importation events into NYC from the broader United States (median = 59; 95% HPD, 43–75), with both importations from and exportations into the United States peaking in late June to early July (Fig. 2a). However, there were many exportations of MPXV from NYC to the rest of the United States (median = 170; 95% HPD, 143–193; Fig. 2b).

**Extended Data Fig. 1:**
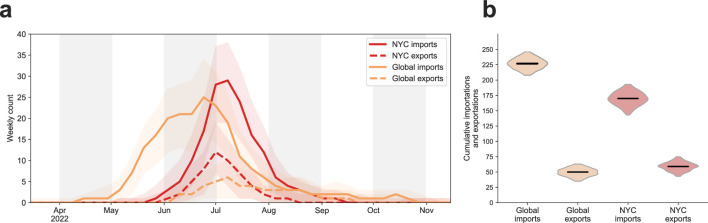
Importations and exportations of MPXV in the United States. **a**, The estimated number of MPXV importations into and exportations out of the United States per week. Center line indicates median value, and shaded areas show 95% highest posterior densities (HPDs) of the estimates shaded areas show 95% highest posterior densities (HPDs) of the estimates. **b**, Cumulative number of MPXV importations into and exportations out of the United States. The violins represent the 95% HPDs and the black bars indicate the median values.

Global importations seeded the start of the epidemic in the rest of the United States (Extended Data Fig. 1). Then, starting in July, most importations into the broader United States came from NYC. By the end of 2022, there were nearly as many importations from NYC into other areas of the United States as from the rest of the world.

### MPXV transmission clusters

**Extended Data Fig. 2:**
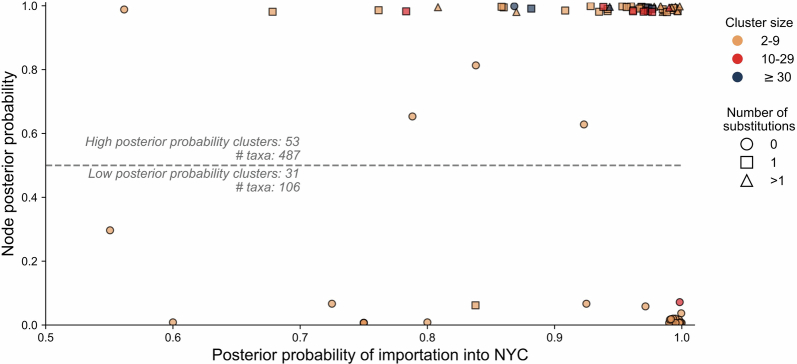
Posterior probability of importations. The posterior probability of each importation cluster, excluding singletons, compared against its posterior probability of being imported into NYC. Note that each importation event must appear as an importation in at least 50% of the posterior samples. Points are colored by cluster size, and their shapes indicate the number of substitutions separating the import from its parent node in the mutation tree. Jitter has been applied at posterior probability values of 1 to make individual points more visible.

**Extended Data Fig. 3:**
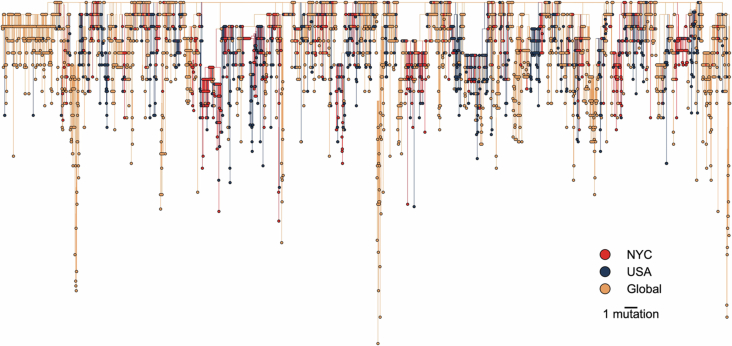
Mutation tree of MPXV. Branches are colored based on locations inferred from the phylogeography.

**Extended Data Fig. 4:**
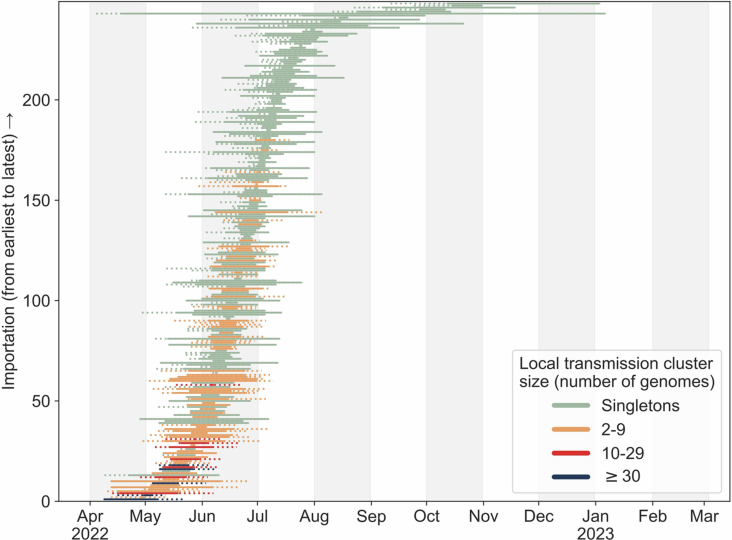
Estimated importation times for NYC transmission clusters, including singletons. The left limit of the solid line is the median time of the parent node of the MRCA of each transmission cluster and the right limit is the median tMRCA of the transmission cluster. The left limit of the dotted line is the lower bound of the 95% HPD of the time of the parent node and the right limit is the upper bound of the 95% HPD of the tMRCA. For singletons, the right limit of the solid line is the date of sampling, and there is no dotted line extending rightward.

Our phylogeographic analysis allows for the identification of NYC transmission clusters: distinct importations of MPXV into NYC that result in identifiable onward transmission within NYC (that is, at least two individuals with closely related sequenced MPXV genomes). We inferred 84 clusters in total, and many of these clusters could be reliably identified (Extended Data Fig. 2), because they often were defined by at least one substitution (Extended Data Figs. 2 and 3). We can be reasonably confident in these phylogenetic relationships because the underlying mutation tree falls in the 'near-perfect' tree space: it has a short overall length (0.0291 substitutions per site) in which most nodes are defined by only one or two mutations, leading to an expected false-positive rate for any internal node of 0.0097 (ref. ^30^). The importations of MPXV into NYC that led to the largest transmission clusters are estimated to have occurred in late April or early–mid-May 2022 (Fig. 2c). However, most imports, especially later in the outbreak, led to singletons: introductions characterized by a single viral genome without any identifiable onward transmission in NYC (Extended Data Fig. 4).

We assessed the association between demographic attributes obtained during interview with NYC DOHMH epidemiologists and MXPV transmission risk reported to public health surveillance, and the frequency of membership in NYC MPXV transmission clusters among 699 individuals (699/756; 92.5%) for whom this information was reported. This population mostly consisted of MSM (Table 1). We observed increased odds of membership in an MPXV transmission cluster for Black or African American individuals (*P* = 0.038) relative to Latino/Latina or Hispanic individuals, whereas white individuals (*P* = 0.005) had decreased odds of clustering. We also observed decreased odds of MPXV clustering for people reporting recent foreign travel history (*P* = 0.001). An explanation for this inferred negative association between clustering and foreign travel is found in our definition of clusters as unique introductions into NYC. Although each transmission cluster must have, by definition, been initiated by someone with recent travel history, introductions that did not lead to onward transmission would include only an individual with foreign travel history. (Fig. 2d and Extended Data Fig. 4). Membership in a cluster did not vary significantly by gender, sexual orientation, age or vaccination status.

**Table 1:** Attributes associated with membership in MPXV clusters using multivariate logistic regression modeling

Attribute	Category	Cases	Clustered		Adjusted OR	OR 95% CI	*P*-value^a^
			*n*	%			
All	–	699	549	78.5	–	–	–
Gender	Cis-male	681	533	78.3	2.11	0.56–13.75	0.337
	Trans-woman/Nonbinary	18	16	88.9	Ref.	–	–
Sexual orientation	Bisexual	50	40	80.0	0.68	0.32–1.56	0.340
	Gay	492	379	77.0	Ref.	–	–
	Heterosexual	31	25	80.6	0.60	0.24–1.74	0.307
	Unknown/Other	126	105	83.3	1.28	0.73–2.33	0.401
Borough	Bronx	120	101	84.2	1.29	0.71–2.41	0.412
	Brooklyn	161	141	87.6	2.21	1.27–4.00	0.006
	Manhattan	309	223	72.2	Ref.	–	–
	Queens	109	84	77.1	0.97	0.56–1.73	0.927
Foreign Travel	Yes	46	23	50.0	0.30	0.15–0.59	<0.001
	No	286	229	80.1	Ref.	–	–
	Unknown	367	297	80.9	0.89	0.57–1.39	0.614
Race/Ethnicity	Black or African American	198	179	90.4	1.89	1.04–3.53	0.040
	Hispanic or Latino/Latina	218	176	80.7	Ref.	–	–
	White	187	124	66.3	0.48	0.29–0.78	0.004
	Unknown/Other	96	70	72.9	0.61	0.33–1.12	0.108
Age (years)	18–24	36	31	86.1	1.70	0.66–5.31	0.312
	25–34	303	230	75.9	Ref.	–	–
	35–44	246	191	77.6	1.27	0.83–1.94	0.277
	45–54	82	69	84.1	1.65	0.86–3.37	0.149
	55+	32	28	87.5	2.57	0.91–9.31	0.105
Vaccination	Yes	42	32	76.2	0.87	0.40–2.00	0.726
	No	657	517	78.7	Ref.	–	–

OR, odds ratio; CI, confidence interval; Ref., reference category.

^a^Two-sided.

### Associations between MPXV and HIV transmission clusters

The distribution of MPXV transmission cluster sizes is highly right-skewed (Fig. 2d), matching previously described patterns of HIV transmission cluster size distributions among MSM^19–21^. To better characterize these MPXV transmission cluster sizes, we fit a set of distributions to them and found that scale-free distributions provided the best fit (Akaike information criterion < 550; Extended Data Table 1). To validate the fit of these distributions, we simulated transmission cluster sizes from them and calculated how similar the simulated cluster sizes were to the inferred cluster sizes. Scale-free distributions consistently fit the inferred MPXV transmission cluster sizes better than distributions that were not scale free, with a Yule–Simon distribution providing the best fit (Kullback–Leibler [KL] divergence = 0.336).

**Extended Data Fig. 5:**
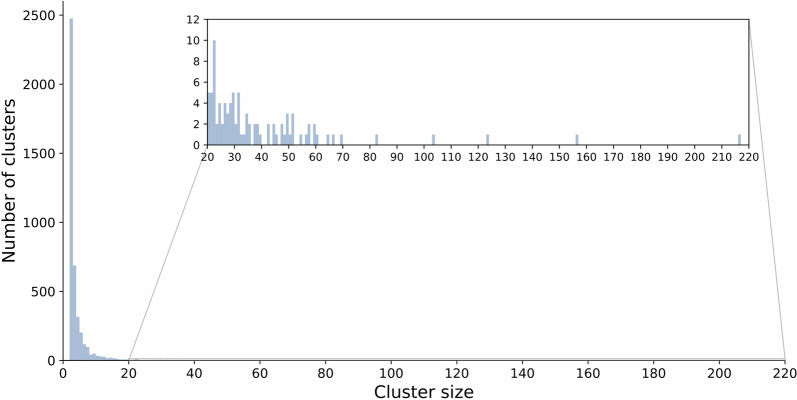
HIV transmission cluster sizes. Inset shows frequency of cluster sizes of at least 20.

We then compared the cluster size distributions of MPXV and HIV in NYC. We first inferred transmission clusters from HIV partial*-pol* sequences sampled since 2001 from 76,910 people living with HIV (PLWH) in NYC using HIV-TRACE, identifying 4,259 HIV transmission clusters consisting of 17,456 individuals. We found 416 HIV transmission clusters that grew by at least one member in 2021 or 2022, consisting of 4,097 people. Of these people in growing clusters, 1,711 (40.2%) had a virus with a recent genetic link to another person with an HIV diagnosis in 2021 or 2022. Fitting distributions to the HIV transmission cluster sizes, we found that the HIV transmission cluster sizes in NYC were also right-skewed (Extended Data Fig. 5) and best fit by scale-free distributions (Extended Data Table 1). Both MPXV and HIV transmission clusters in NYC follow scale-free distributions, recapitulating transmission across scale-free sexual transmission networks with heavy-tailed partnership distributions^31^, suggesting that their transmission dynamics were likely to have emerged from similar forms of transmission.

**Extended Data Fig. 6:**
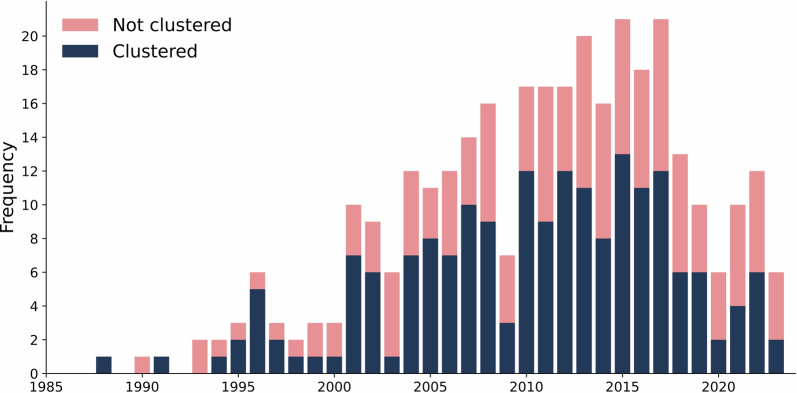
Year of HIV diagnosis for individuals diagnosed with mpox and living with HIV. The bars are colored by whether the individuals are members of mpox clusters.

We queried the HIV surveillance database in NYC to determine whether persons living with HIV and in the HIV transmission network were disproportionately represented in MPXV clusters (Table 2). A substantial proportion of the individuals with an mpox diagnosis had a reported HIV diagnosis (*n* = 328/756; 43.4%), and we found a significant association between a reported HIV diagnosis and membership in MPXV transmission clusters (logistic regression; odds ratio = 1.58; *P* = 0.012). The majority (310/328, 94.5%) of people with both an HIV and mpox diagnosis received their HIV diagnosis prior to 2022, highlighting the different time frame in which these viruses spread among the same people (Extended Data Fig. 6). Individuals who were clustered in both the MXPV network and the HIV network did not share connections in their networks, suggesting that the contact networks for MPXV and HIV were non-overlapping, in line with the different time periods in which individuals received MPXV and HIV diagnoses.

**Table 2:** MPXV and HIV transmission lineage and cluster matching

HIV outcomes	Total (*n*)	Outcome (*n*)	MPXV clustered (*n* = 592)		Not MPXV clustered (*n* = 164)		OR	OR 95% CI	*P*-value
			Yes	No	Yes	No			
HIV diagnosis	756	328	271	321	57	107	1.58	1.11–2.27	0.012^a^
Reported HIV genotype	328	187	155	116	32	25	1.04	0.59–1.86	0.884^a^
HIV clustered	187	96	81	74	15	17	1.24	0.58–2.66	0.580^a^
HIV cluster growth	187	32	31	124	1	31	NA	NA	0.019^b^
HIV recent link	187	15	15	140	0	32	NA	NA	0.078^b^

^a^Logistic regression

^b^Fisher’s exact test; logistic regression was not performed due to restricted sample sizes.

Breakdown of 756 individuals with reported MPXV genomes from PHL included in the phylogeographic analysis based on the clustering profile, association with the HIV transmission network, and related information from the HIV surveillance database. OR, odds ratio; CI, confidence interval; NA, not applicable.

Clustering in the HIV network is associated with more densely connected sexual networks and rapid viral transmission^18,27,32^. We did not observe an association between mpox diagnoses with reporting of HIV sequences or clustering in the HIV transmission network. However, the frequency with which people with an mpox diagnosis clustered in the HIV network (*n* = 96/187; 51.3%) is substantially higher than would be expected; when resampling the HIV network matching transmission risk and diagnosis year of people with an mpox diagnosis, one would expect only 67 individuals (35.8%; 95% quantile range: 55–80) to be clustered in the HIV network. These results indicate that people with both HIV and mpox diagnoses are part of more densely connected sexual networks.

This association of mpox cases with active sexual networks is further demonstrated by their increased occurrence in recently growing HIV transmission clusters (Fisher’s exact test; *P* = 0.019). Nearly all individuals with an mpox diagnosis and membership in a growing HIV cluster (that is, ≥1 new member diagnosed in 2021–2022) were also part of an MPXV cluster (*n* = 31/32). Furthermore, all 15 individuals with an mpox diagnosis and a genetic link to someone diagnosed with HIV in 2021 or 2022 in the HIV transmission network belonged to an MPXV cluster, although the small dataset offers limited statistical power (Table 2).

We next assessed the association between demographic attributes and MXPV transmission risk reported to public health surveillance and the presence of a reported HIV diagnosis among the 699 individuals for whom this information was reported (Extended Data Table 2). We observed lower odds of having a reported HIV diagnosis among individuals identifying as heterosexual (*P* < 0.001), compared with gay men, according to NYC DOHMH interviews. Compared with Latino/Latina and Hispanic people, we observed higher odds for a reported HIV diagnosis among Black and African American individuals (*P* = 0.01) and lower odds for white individuals. Furthermore, all age groups above 34 years old had increasingly higher odds of a reported HIV diagnosis (*P* < 0.001), highlighting the intersection between MPXV and HIV among people who have been living with HIV for years or decades.

### Decline of the NYC mpox outbreak

**Fig. 3:**
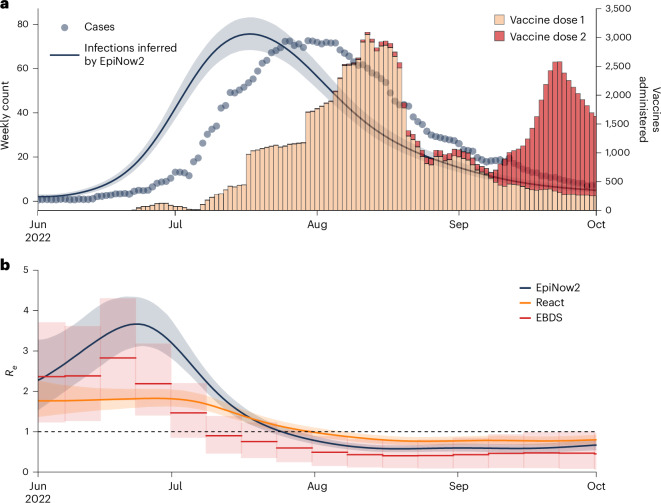
Vaccine administration and effective reproduction number over time. **a**, Number of mpox vaccines administered in mid-late 2022. **b**, Inferred effective reproduction number (*R*_e_) of MPXV based on the case data when incorporating generation time and incubation period into the inference (EpiNow2, blue), solely on the case counts (REACT, yellow), or MPXV genomes in a phylodynamic approach using an episodic birth–death-sampling model (EBDS; red). The dashed line indicates the inflection point (that is, when the epidemic is in decline; *R*_e_ = 1). Lines and shaded regions represent the median and 95% confidence interval, respectively. The EBDS model assumes week-long intervals with a uniform *R*_e_ per interval.

To quantify when and why the 2022 mpox outbreak in NYC began its decline, we inferred the time-varying reproduction number (also known as the effective reproduction number, *R*_e_) using case counts, generation time and incubation period. The epidemic in NYC reached its peak at the end of July 2022 (Fig. 1a), before most of the first vaccine doses of a two-dose vaccine series were given (Fig. 3a). We found that *R*_e_ peaked in late June and began decreasing as the rise in infections slowed and before fewer than 1,000 doses of the first vaccine dose were administered (that is, <1% of the 102,183 first vaccine doses delivered in NYC in 2022) (Fig. 3). Furthermore, *R*_e_ decreased to below 1 by August 2022, before 200 doses of the second vaccine dose were given (that is, <1% of the 52,374 second vaccine doses delivered in NYC in 2022).

Generation time and incubation period can vary throughout an epidemic^33^, thereby resulting in model misspecification. Therefore, we also inferred *R*_e_ using solely case counts, and found that *R*_e_ could have dropped below 1 up to 1 week later (Fig. 3b), but still before a moderate proportion of the second vaccine was given.

The identification of local transmission clusters through our phylogeographic inference provides an alternative approach to measure the changes in *R*_e_ during the NYC mpox epidemic that relies on genomics and inferred local transmission. We constructed an episodic birth–death-sampling model in a Bayesian framework that jointly analyzed all imported NYC clusters with at least 10 genomes (*n* = 13). We found that *R*_e_ decreased below 1 by mid-July, approximately 1–2 weeks before the dates inferred by primarily using case data, in line with many of the introductions of MPXV into NYC after June that did not have identifiable onward transmission (Extended Data Fig. 4). Additionally, across all models, *R*_e_ had declined to near 1 by the time that infections probably peaked and fell below 1 by the time that reported cases began declining (Fig. 3).

**Extended Data Fig. 7:**
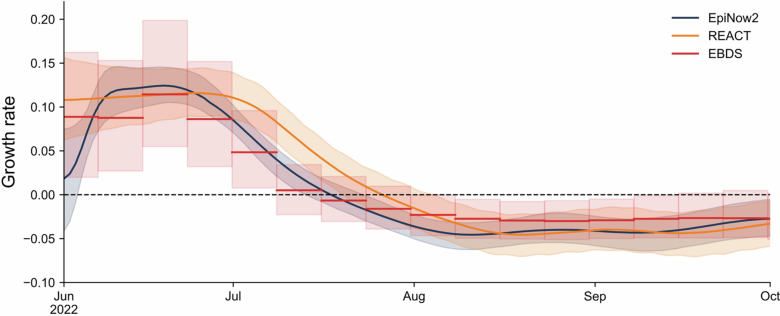
Effective reproduction number and growth rate of MPXV. Inferred growth rate of MPXV based on the case data whilst incorporating generation time and incubation period into the inference (EpiNow2, blue), solely on the case counts (REACT, yellow), or MPXV genomes in a phylodynamic approach using an episodic birth-death-sampling model (EBDS; red). Dashed line indicates inflection point (*that is*, when the epidemic is in decline; growth rate=0). Lines and shaded regions represent the median and 95% confidence interval, respectively. The EBDS model assumes week-long intervals with a uniform growth rate per interval.

To confirm these results, we inferred the growth rate of mpox (Extended Data Fig. 7), given that growth rate has been shown to be less sensitive than *R*_e_ to model misspecification^34^. The growth rate also began declining by the end of June, when fewer than 1,000 doses of the first vaccine dose had been given, and decreased below zero by August. Growth rate inference was consistent both with and without the generation time and incubation period parameterization. When focusing on local transmission clusters, we again found that the growth rate decreased below zero approximately 1–2 weeks prior to the inferred dates when using the other approaches.

### Heavy-tailed sexual contact network drives outbreak dynamics

**Extended Data Fig. 8:**
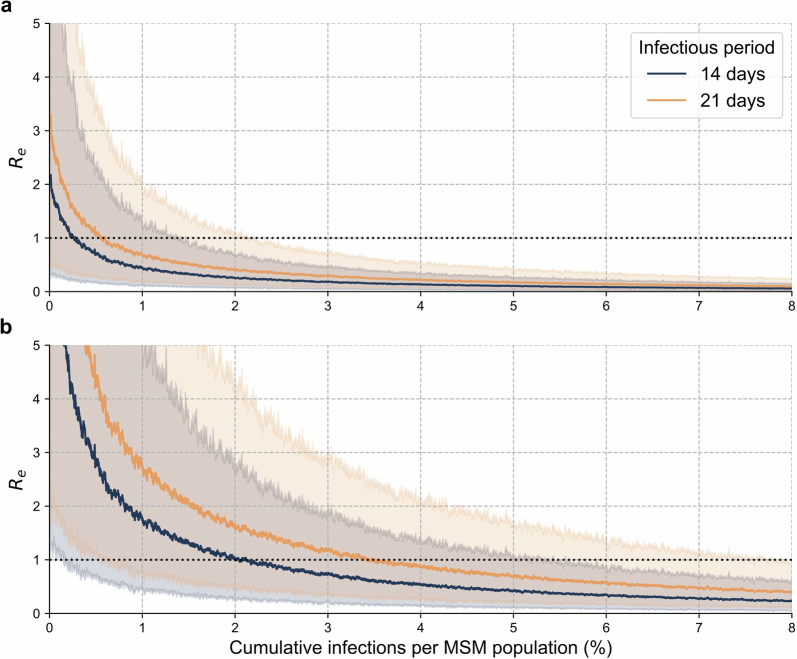
Effective reproduction number and depletion of highly connected susceptibles of simulated MPXV epidemics. The effective reproduction number (R_e_) after observing given cumulative infections with varying infectious periods and an SAR of (**a**) 0.1 and (**b**) 0.4. Lines and shaded regions represent the median and 95% quantile range, respectively.

To determine whether a heavy-tailed sexual contact network could explain the decline of the outbreak before there was substantial infection- or vaccine-derived immunity, we simulated mpox epidemics based on MSM sexual contact networks, under varying assumptions for the infectious period and the risk of transmission between sexual partners (secondary attack rate [SAR] during the infectious period). By the time *R*_e_ dropped below 1 in our simulations, we estimated a cumulative incidence among MSM of below 2% when assuming an SAR of 0.1 (Extended Data Fig. 8a); a higher SAR produces slightly higher average cumulative incidence, but with a larger range (Extended Data Fig. 8b). Assuming an MSM population in NYC of approximately 235,000 (Methods), we observed an empirical cumulative incidence of 0.52–0.96% (*n* = 1,230–2,248) at the time *R*_e_ dropped below 1 (Fig. 3). If we conservatively assume a 60% mpox case ascertainment rate^4,35^, there would have been an empirical cumulative incidence of 0.87–1.59% at this time. These results are broadly consistent with our simulations, suggesting that the dynamics of the mpox outbreak in NYC were likely governed by a heavy-tailed sexual network, with an SAR between 0.1 and 0.4 (Extended Data Fig. 8).

**Fig. 4:**
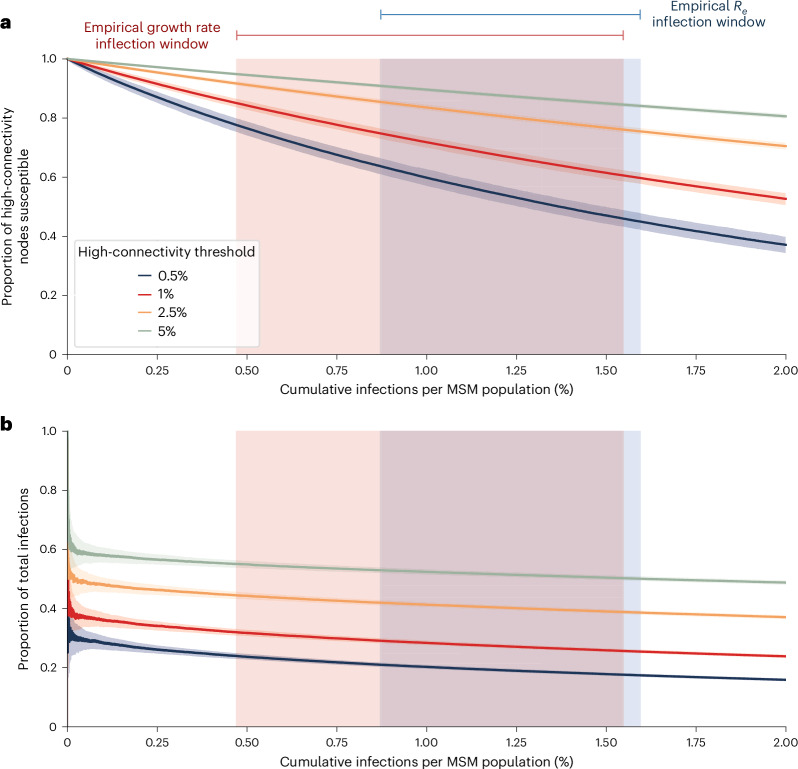
Infection of highly connected individuals. **a**, Proportion of highly connected susceptible individuals remaining after observing given cumulative cases. **b**, The proportion of cumulative infections, consisting of infected highly connected individuals. The high-connectivity threshold refers to the top percentile of network connectivity. Lines and shaded regions represent the median and 95% quantile range, respectively. The red and blue blocks represent when the inferred growth rate and *R*_*e*_ reached their inflection points based on the empirical data. The left and right limits of the blocks are, respectively, the lowest lower limit and the highest upper limit across the 95% confidence intervals of the methods based on case counts detailed in Fig. 3 and Extended Data Fig. 7.

From these epidemic simulations, we found that the most highly connected individuals (that is, people with more sexual partners than at least 99% of the sexual contact network) become infected early in the epidemic and nearly 40% of these individuals were infected by the time the empirical *R*_e_ dipped below 1 (Fig. 4a). This dynamic results in more than 25% of all infections consisting of highly connected individuals by the time *R*_e_ declined to 1 (Fig. 4b); across varying connectivity thresholds, a disproportionate number of the cumulative cases are attributed to the highly connected individuals. The rapid infection of highly connected individuals in the sexual network at the start of the epidemic suggests that their connectivity was a major contributor to the initially high *R*_e_ and that the early saturation of densely connected parts of the sexual network contributed to the epidemic ending with low cumulative incidence.

## Discussion

Long-standing molecular surveillance in NYC provided an opportunity to investigate the associations between the MPXV and HIV transmission networks and whether MPXV recapitulated the dynamics of a sexually transmitted pathogen such as HIV. We found that earlier introductions of MPXV into NYC resulted in, on average, larger transmission clusters than later introductions, following model-based expectations^36^, and that the largest clusters were likely to have been seeded before the first dozen cases were identified in NYC. Our comparative analysis of MPXV with HIV and epidemic modeling showed that transmission through a heavy-tailed sexual contact network can explain the epidemic trends observed in NYC.

Sexual transmission networks, particularly among MSM, are characterized by heavy-tailed degree centrality distributions^14,37,38^, which are also observed in HIV transmission networks^20,21^. Considering the concordance between HIV and MPXV transmission networks and the similarities in their cluster size distributions, the MPXV sexual network is likely to be a heavy-tailed partnership distribution as well. The extent to which these MPXV and HIV cluster-size distributions truly represent scale-free contact networks is a matter of ongoing debate^39,40^. However, cluster size distributions depend on the structure of the contact network, and heavy-tailed degree distributions are known to produce power-law cluster size distributions^31^.

Epidemic simulations developed to predict and explain the course of the 2022 mpox epidemic assumed a heavy-tailed transmission network, based on previously characterized sexual networks^14,15^. By using genomic epidemiology and building upon these simulations, we confirmed this assumption is valid and demonstrated that the 2022 mpox outbreak in NYC was consistent with the rapid and early infection of highly connected individuals in the sexual contact networks. Although these highly-connected individuals make up a small proportion of the overall contact network, their immunity, whether from vaccination or natural infection, would disrupt chains of transmission. The decrease in transmission cluster sizes as the outbreak progressed is therefore likely the result of infection-derived immunity of these highly connected individuals, when fewer than 2% of the at-risk MSM population are believed to have been infected^15^.

Both MPXV and HIV spread through heavy-tailed sexual contact networks in NYC, primarily consisting of MSM. However, the differing natural histories and evolutionary histories of these viruses produced transmission networks that intersected, but did not recapitulate each other. The preponderance of HIV diagnoses among MPXV cases were from the 1990s and 2000s, resulting in older individuals being disproportionately infected with both viruses. Hence, people living with HIV acquired MPXV from sexual partners who were not members of their original HIV transmission cluster. In the years separating HIV and MPXV transmission events, sexual contacts are likely to have shifted, while an overall scale-free sexual contact network structure was maintained. Although we conclude that the structure of this network led to the decline of the 2022 mpox outbreak in NYC, the saturation of this network has not produced a concomitant decline in HIV cases. The ability of HIV to produce chronic infections and transmit over years and decades in this same population suggests that shifting sexual contact networks are key to its persistence. Future work on the role and timing of sexual network restructuring may improve long-term prevention strategies for HIV and other sexually transmitted pathogens.

Contact network heterogeneity, changes in behavior, and vaccines could each individually affect MPXV transmission dynamics. Although there were undoubtedly changes in sexual behavior globally after the World Health Organization (WHO) declared mpox a public health emergency of international concern (PHEIC) on 23 July 2022^41–43^, these changes would have occurred after *R*_e_ was already near or below 1 and therefore could not have led to the decline of the NYC outbreak. Instead, the behavioral changes in NYC that would have contributed to limit the transmission of MPXV are likely to have occurred early in the 2022 outbreak^44–47^. A previous genomic epidemiological investigation found that vaccination had limited impact in curbing the North America mpox outbreak, with fewer than 10% of high-risk individuals developing vaccination-induced immunity before *R*_e_ reached 1. By coupling genomic epidemiology with epidemic modeling and focusing on NYC, which experienced the largest outbreak in North America and one of the earliest vaccine rollouts, we underscore the limited role that vaccination played in curbing the 2022 mpox epidemic and provide a mechanistic explanation for its trajectory.

Although NYC rapidly distributed mpox vaccines to those at greatest risk of infection, the rise and fall of the epidemic appears to have outpaced the delivery of the vaccine. Mpox vaccination requires two doses 1 month apart, and immunocompetent individuals are considered fully immunized 2 weeks after the second dose^48,49^. A substantial fraction of at-risk people in NYC did not reach this point until September 2022, suggesting that the mpox vaccination campaign most probably occurred too late to substantively affect the trajectory of the NYC mpox outbreak. Even though the mpox vaccination campaign was probably not responsible for slowing and ultimately curbing the 2022 epidemic, vaccination – even a single dose – is likely to have protected many individuals from infection and severe disease^50–53^. Importantly, the lack of a resurgent mpox outbreak in NYC in the subsequent year, despite the continued circulation of MPXV among MSM^7^, suggests that vaccination may have played a role alongside natural (infection-induced) immunity in preventing resurgent community transmission.

These results should be interpreted in the context of several limitations. First, uneven sampling and sequencing coverage could bias the inference of viral importations and onward transmission. Specifically, genomic sampling peaked in NYC before the cases did (Fig. 1a,b); however, considering that NYC has the highest proportion of cases sequenced, it is unlikely that we overestimated the number of introductions of MPXV into NYC. Nearly all of the sequenced NYC cases were from sexual health clinics, which, although not necessarily representative of citywide cases, are likely to represent a sentinel population with which to understand sexually transmitted pathogens in NYC. Second, generation times, serial intervals, and delays to diagnosis of MPXV probably varied over the course of the epidemic, as evidenced by different estimates across regions and timeframes^54–58^. We therefore inferred the growth rate in addition to the effective reproduction number and used multiple approaches for the inference of each, arriving at consistent results for when the outbreak ended. Last, for our simulations, we restricted the population to MSM, assumed that the sexual partnership distribution estimated for the MSM population in the United Kingdom was a sufficient proxy for that in NYC, and did not account for network structure or changes in behavior. However, heterosexual transmission of MPXV has not been shown to be a major factor in the 2022 mpox epidemic, and a more heterogeneous population than the one modeled or substantive shifts in behavior could result in an epidemic that could end with even lower cumulative incidence.

The WHO declared an end to mpox as a PHEIC in May 2023; however, there have since been numerous outbreaks of MPXV, specifically clade Ia and clade Ib in west and central Africa, as well as continued transmission of clade IIb throughout the world. As a consequence of the clade I outbreaks and an upsurge of mpox in the Democratic Republic of the Congo and other countries in Africa, the WHO again declared mpox a PHEIC in August 2024. These 2024 outbreaks have impacted a broader demographic than the 2022 epidemic, including an increased proportion of cases among children and sex workers^59,60^, and could indicate that MPXV is capable of spreading across differently structured contact networks. It is therefore critical to deploy targeted and rapid vaccination strategies to mitigate current and future mpox outbreaks.

## Methods

### Ethics review

The activities pertaining to this study at NYC DOHMH are routine analysis of public health surveillance data and thus not subject to institutional review board (IRB) approval. The IRB of the University of California San Diego determined the MPXV and HIV analysis to be of minimal risk and as not qualifying as human subjects research per the Code of Federal Regulations, Title 45, part 46, and the UC San Diego Standard Operating Procedures and Policies.

### Incidence and vaccine data

The number of reported mpox cases per country was downloaded from Our World in Data (OWID; https://ourworldindata.org/; last accessed on 6 February 2024).

The number of reported mpox cases and vaccines administered in NYC were generated by the NYC DOHMH (https://github.com/nychealth/monkeypox-data and https://github.com/nychealth/mpv-vaccine-data?tab=readme-ov-file, respectively; last accessed on 6 February 2024).

### Genomic data and initial processing

We generated 1,144 MPXV sequences from 759 individuals as part of public health surveillance by the NYC DOHMH PHL^61^, which are available for download on NCBI as part of BioProject PRJNA949682.

We downloaded all available MPXV sequences from GISAID (Global Initiative on Sharing All Influenza Data; last accessed 23 May 2023) while excluding the NYC sequences matching ours from NCBI. From these genomes, we subsequently excluded any genomes sampled before 28 April 2022 or after April 2023 and those that did not belong to the clade II, B.1 lineage, resulting in one additional genome from NYC, 1,221 from the rest of the United States, and 2,338 from the rest of the world. A 2021 Maryland sample (NCBI accession number ON676708) was added to this dataset for use as an outgroup.

Genomes with fewer than 180,000 non-'N’ sites were subsequently excluded. We aligned the remaining MPXV genomes against the clade II reference genome (NC_063383) and masked the inverted terminal repeat regions, as well as repetitive and low complexity regions, using squirrel^62^ (https://github.com/aineniamh/squirrel). Given that some regions of a subset of genomes were still poorly aligned, we removed stretches of sequences with many Ns. Specifically, we iteratively examined each sequence using a window of size 100 nucleotides, and if the start and end of the window were each an N and more than ≥25% of the window were Ns, we masked the given window. Genomes that had either fewer than 10 (*n* = 1) or more than 90 (*n* = 10) substitutions relative to the reference genome were considered outliers and were excluded.

Sequences from individuals from NYC with multiple high-quality genomes were filtered to include only the earliest genome from each individual, and if there were multiple genomes with the same collection date for a given individual, the genome with the fewest ambiguities was chosen. The final dataset consisted of 4,044 genomes, with 757 genomes from NYC (756 generated by PHL), 1,127 from the broader United States, and 2,160 from the rest of the world.

The percentage of cases sequenced is based on the number of cases from OWID and the number of genomes from each region from 28 April 2022 to 28 April 2023. The number of genomes was calculated before filtering, with the exception of only counting each case from NYC with multiple genomes once.

The accession IDs for all genomes used can be found in Supplementary Table 1 (all) and Supplementary Table 2 (GISAID).

### Phylogenetic analysis

We inferred a maximum likelihood tree with MAPLE v0.3.3 (ref. ^63^) using a general time-reversible model. We extracted the B.1 clade subtree and then converted it from a bifurcating tree to a multifurcating tree using the ape package^64^ in R v4.3.2 (ref. ^65^).

We performed time-calibration of the resulting multifurcating tree using a recently implemented model in BEAST v1.10 (ref. ^66^), which replaces the traditional likelihood with a more efficient likelihood approximation^67^ based on a Poisson model. In this approach, a starting tree scaled to substitutions per site is provided and the tree search is constrained such that only node heights and polytomy resolutions are sampled. We used a starting clock rate of 5.0 × 10^−5^ and a relatively uninformative gamma prior with a shape of 0.001 and scale of 1,000, as well as a nonparametric coalescent tree^68^ prior with 26 evenly placed grid-points between the most recently sampled sequence and 2 years prior to that time. We simulated 15 Markov chain Monte Carlo (MCMC) chains of 5 × 10^8^ to 1 × 10^9^ generations, subsampling every 25,000 iterations to continuous parameter log files and every 250,000 iterations to continuous tree files. The first 10–20% of each chain was discarded as burn-in for the analyses, and the chains were then combined using LogCombiner. The resulting parameter log and tree files were resampled every 500,000 and 5,000,000 states, respectively. Model convergence and mixing was assessed in Tracer v1.7.1 (ref. ^69^), and all effective sample sizes were >150.

To reconstruct the importation dynamics of MPXV, we used an asymmetric discrete-trait analysis model^70^ implemented in BEAST v1.10 with samples assigned to NYC, United States (excluding NYC), and global (excluding the United States in its entirety) locales. We used the resampled empirical tree distribution described above and simulated one MCMC chain of 1 × 10^6^ generations, subsampling every 100 iterations to a continuous parameter log file and every 500 iterations to a continuous tree file. We discarded the first 10% of the chain as burn-in and resampled the trees every 1,500 states. A maximum clade credibility (MCC) tree was then generated with TreeAnnotater v1.10 using the subsampled trees. For individual posterior tree samples, an introduction into a region is defined when a node is assigned to a particular location with high probability from the posterior distribution and its parent node has a different location^71^. For the MCC tree, we define an introduction into a region when a node has a posterior probability >0.5 for the given location and its parent node has a posterior probability of ≤0.5 for the same location. Based on an established approach to identify the cluster sizes resulting from an introduction^36,72^, we performed a depth-first search starting at all internal nodes that correspond to an introduction of MPXV, traversing forward in time until a node corresponding to a different region (that is, an exportation) is encountered or there are no more nodes remaining to be explored. Transmission clusters are defined as introductions that led to at least two genomes, and introductions resulting in one genome are labeled singletons. When showcasing the time of importation for each transmission cluster, we include the entire 95% HPD intervals of the time of the most recent common ancestor (MRCA) of the transmission cluster and the time of the parent node of this MRCA.

We next sought to determine how many identifying mutations preceded each imported transmission cluster. Given that the only difference between the mutation tree and the time tree topologies is in the resolution of polytomies, we are able to convert the MCC tree from BEAST into a mutation tree with analogously resolved polytomies. Using the same approach described earlier except with this mutation tree, we identified introductions into NYC and the size of the imported transmission clusters (note that these are identical as when determined using the MCC tree). We then inferred the branch length leading to the MRCA of each transmission cluster in terms of the inferred number of substitutions per site. We rounded the number of inferred substitutions to the nearest whole number (Extended Data Fig. 2).

We further explored the transmission dynamics of MPXV through the application of an episodic birth–death sampling (EBDS) model in BEAST. This phylodynamic approach enables the investigation of dynamic changes in birth, death and sampling rates of individuals over discrete time epochs^73^. We conducted a joint phylogeny analysis, in which we inferred conditionally independent trees for all clusters consisting of at least 10 genomes (*n* = 13), all derived from the same EBDS model.

We implemented an EBDS model with a cut-off of 10 months, inferring time-varying birth and sampling rates across 40 epochs, each representing 1 week. The model assumes a constant death rate over time, no intensive sampling events at epoch transitions, and the removal of lineages upon sampling. Gaussian Markov random field priors are used for the birth and sampling rates to accommodate the substantial variability in effective reproduction number over time. The prior distribution for the constant death rate follows a log-normal distribution, with mean and standard deviation values estimated to yield a 95% confidence interval for infectious duration from 2 to 4 weeks.

For tree inference, we applied a general time-reversible nucleotide substitution model for all trees and a strict molecular clock model for each cluster, incorporating continuous-time Markov chain reference priors^74^ for the evolutionary rates.

We simulated 10 MCMC chains of 1 × 10^9^ generations each, facilitated by Hamiltonian Monte Carlo transition kernels^75^, and subsampling every 1 × 10^5^ iterations to continuous parameter log files. The first 10% of each chain was discarded as burn-in for the analysis, the resulting parameter log files were resampled every 1 × 10^6^ states, and the chains were then combined using LogCombiner.

The effective reproduction number for each epoch is computed as

$${R}_{{\rm{e}}}({\rm{t}})=\frac{\lambda \left(t\right)}{\mu \left(t\right)+\psi (t)}$$

and the growth rate is calculated by

$${\rm{r}}({\rm{t}})=\lambda \left(t\right)-\mu (t)$$

where λ, μ and *ψ* are functions of time (*t*) representing the birth rate, death rate and sampling rate, respectively.

Phylogenetic trees were processed using TreeSwift v1.1.42 (ref. ^76^) and visualized using baltic v0.2.2 (ref. ^77^).

### Demographics analysis

Individual demographic and risk characteristics were abstracted by NYC DOHMH from medical records, which were later updated by NYC DOHMH epidemiologists during interviews. Correlates of membership in MPXV transmission clusters were assessed using logistic regression in R v4.0.2. Of the 756 people with a high-quality sequenced MPXV genome and in the PHL surveillance network, demographic and transmission risk metadata were available for 708 individuals. To achieve statistical convergence, we excluded all groups with fewer than 10 individuals (that is, 2 cis-women, 3 individuals with unknown gender, and 4 individuals residing in Staten Island) from our regression analysis, resulting in 699 included individuals.

### HIV transmission network

Because HIV has been circulating in NYC since the 1970s and we are interested in the comparison of more recent transmission clusters, we used HIV-TRAnsmission Cluster Engine (HIV-TRACE)^78^ to infer these clusters based on genetic distance between these virus genomes, rather than identifying clusters using phylogeographic inference. The first reported sequence for each person was aligned to the HXB2 reference PR + POL sequence (positions 2253–5094) and trimmed to PR/partial-RT to positions 2253–3749. Sequences with ≥5% ambiguous nucleotides and those with ≤0.015 substitutions per site from the HXB2 reference sequence were excluded. Pairwise TN93 genetic distances was compared among the first reported viral sequences from 76,910 individuals residing in or receiving HIV care in NYC reported to the NYC DOHMH as of January 2024; an HIV transmission network was constructed using a distance threshold of 0.015 substitutions per site (resolving distances between ambiguous nucleotides for sequences with ≤1.5% ambiguities). For each clustered individual, we determined, first, whether their cluster had added a case diagnosed in 2021; and second, if they were directly genetically linked to a virus from a case diagnosed in 2021.

Matching between individuals with an MPXV and HIV diagnosis reported to DOHMH was performed based on a 36-key deterministic matching algorithm^79^. The relationship between MPXV cases and HIV cases reported to the NYC DOHMH was assessed using logistic regression when expected values were ≥5 and Fisher’s exact test when expected values were <5.

### Transmission lineage size distribution fitting

We fit the following distributions to the MPXV transmission cluster sizes using Python v3.10.10: exponential, binomial, negative binomial, Pareto, power law and Yule–Simon. We used a minimum cluster size of 2 and truncated the probability distribution to the minimum cluster size. Fit was determined using the Akaike information criterion. Because much of the probability distribution was typically truncated during the fitting, to further examine which distributions best fit the transmission cluster sizes, we (1) subsequently simulated 10,000 transmission cluster sizes using the fit distributions; (2) we truncated the simulated data to the minimum cluster size; (3) we normalized the inferred and simulated cluster sizes based on the total number of cluster sizes for each; and (4) we calculated the Kullback–Leibler divergence between these two sets of normalized cluster sizes. We repeated this process for the HIV transmission cluster sizes.

### Epidemiology

To supplement the phylogenetic analyses (see above) and characterize the growth and decline of the MPXV epidemic in NYC using more traditional epidemiological approaches, we inferred the time-varying reproduction number (also known as the effective reproduction number, *R*_e_) and the growth rate using EpiNow2 v1.5.2 (ref. ^80^), assuming a gamma-distributed generation time with a mean of 12.6 days, a standard deviation of 5.7 days and a maximum of 25 days, and a gamma-distributed incubation period with a mean of 9.1 days, a standard deviation of 3 days and a maximum of 20 days. We ran four chains for 2,000 iterations each, with a warmup of 400 iterations, a maximum tree depth of 12, and an average proposal acceptance probability ('adapt_delta') of 0.999 for the No-U-Turn Sampler^81^ used by Stan^82^. Given that the generation time and incubation period can vary over the course of an epidemic^33^, we also calculated *R*_e_ and growth rate using the approach from ref. ^83^, which uses only case counts from the epidemic. For this approach, we ran four chains for 5,000 iterations each, with a warmup of 1,000 iterations, a maximum tree depth of 20 and an average proposal acceptance probability of 0.999. Note that the package from ref. ^81^ needed to be manually adjusted to enable the changing of maximum tree depth and average proposal acceptance probability. The time periods in which the effective reproduction number and growth rate reached their inflection points shown in Fig. 4 were calculated using the two approaches described here.

### Simulating epidemics

We simulated mpox epidemics using Julia v1.10.2 based on the approach in ref. ^14^. In brief, this approach uses a branching process model of transmission among an MSM population. The MSM sexual network was characterized by a Weibull distribution with parameters that were estimated by fitting a Weibull distribution to the empirical degree distribution from the Natsal dataset^84–86^, which is composed of MSM who had at least one sexual partner over the course of 1 year. The simulations assumed a single starting case and in each case the virus was transmitted to their sexual partners with a constant probability per partner (that is, secondary attack rate [SAR]) over the infectious period.

To determine the size of the MSM population in NYC in 2022 for the simulations, we multiplied the proportion of MSM per county in NYC based on ref. ^87^ by the expected number of adult men living in each county^88^. We used the reported proportions of MSM for each county in NYC, but we used the average New York State proportion of MSM for Richmond County (Staten Island) because it was not listed in ref. ^87^. This resulted in an MSM population of approximately 235,000.

We performed 1,000 simulations with this population size, SARs of 0.1 and 0.4, and infectious periods of 14 and 21 days. The effective reproduction number was calculated at each infected case as per ref. ^14^. High-connectivity individuals were defined as those with a degree above a certain percentile (for example, 95th percentile, or the top 5% of connected individuals). We then calculated the proportion of high-connectivity susceptible people remaining as each case was infected.

### Reporting summary

Further information on research design is available in the Nature Portfolio Reporting Summary linked to this article.

## Online content

Any methods, additional references, Nature Portfolio reporting summaries, source data, extended data, supplementary information, acknowledgements, peer review information; details of author contributions and competing interests; and statements of data and code availability are available at 10.1038/s41591-025-03526-9.

## Supplementary information

Reporting Summary

Supplementary Tables 1–2 Accession numbers of genomes used in this study.

## Figures and Tables

**Extended Data Table 1:**
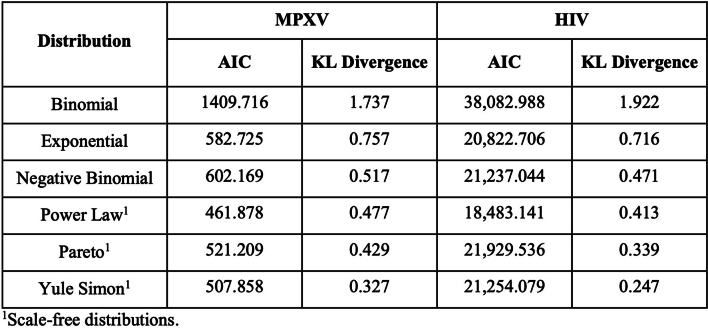
Distributions fit to MPXV and HIV transmission clusters

**Extended Data Table 2:**
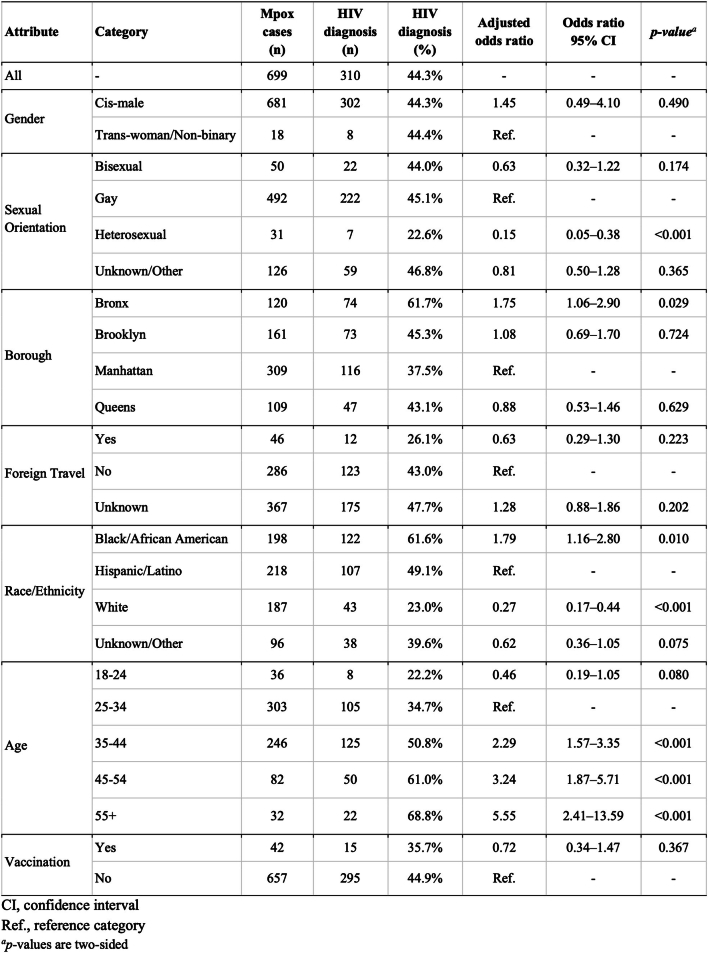
Attributes associated with mpox cases and reported HIV diagnosis using multivariate logistic regression modeling

## Data Availability

MXPV genomes from the NYC DOHMH PHL that the authors generated have been deposited at NCBI (BioProject PRJNA949682). The accession IDs for all MPXV genomes used can be found in Supplementary Table 1 (all) and Supplementary Table 2 (GISAID). The genomes available at NCBI are public; those at GISAID require registration with GISAID. HIV surveillance activities are protected by New York State Redisclosure Law Articles 21 and 27-F, which prevents the submission of HIV-1 genetic sequences to public databases. Data were shared with investigators under data use agreements with UC San Diego. Data requests can be made by emailing ftaki@health.nyc.gov, with an expected time for response of 1 week. Time to provide the data depends on the data request and the potential need of a data use agreement.

## Extended data

are available for this paper at 10.1038/s41591-025-03526-9.

## Code availability

Code is available at https://github.com/pekarj/nyc_mpox_phylogeography.
