# Supplementary material for: Transmission dynamics of the 2022 mpox epidemic in New York City
Source: Nat Med. 2025 Mar 25;31(5):1464–73. doi: 10.1038/s41591-025-03526-9 (PMC12092259; doi:10.1038/s41591-025-03526-9)
Supplement: Supplementary file 1 — Reporting Summary [file 41591_2025_3526_MOESM1_ESM.pdf]

nature portfolio

Corresponding author(s): Jonathan Pekar, Joel Wertheim

Last updated by author(s): Dec 21, 2024

Reporting Summary

Nature Portfolio wishes to improve the reproducibility of the work that we publish. This form provides structure for consistency and transparency in reporting. For further information on Nature Portfolio policies, see our Editorial Policies and the Editorial Policy Checklist.

Statistics

For all statistical analyses, confirm that the following items are present in the figure legend, table legend, main text, or Methods section.

n/a	Confirmed	
☐	☒	The exact sample size (*n*) for each experimental group/condition, given as a discrete number and unit of measurement
☒	☐	A statement on whether measurements were taken from distinct samples or whether the same sample was measured repeatedly
☐	☒	The statistical test(s) used AND whether they are one- or two-sided<br>*Only common tests should be described solely by name; describe more complex techniques in the Methods section.*
☐	☒	A description of all covariates tested
☒	☐	A description of any assumptions or corrections, such as tests of normality and adjustment for multiple comparisons
☐	☒	A full description of the statistical parameters including central tendency (e.g. means) or other basic estimates (e.g. regression coefficient) AND variation (e.g. standard deviation) or associated estimates of uncertainty (e.g. confidence intervals)
☐	☒	For null hypothesis testing, the test statistic (e.g. *F*, *t*, *r*) with confidence intervals, effect sizes, degrees of freedom and *P* value noted<br>*Give P values as exact values whenever suitable.*
☐	☒	For Bayesian analysis, information on the choice of priors and Markov chain Monte Carlo settings
☐	☒	For hierarchical and complex designs, identification of the appropriate level for tests and full reporting of outcomes
☒	☐	Estimates of effect sizes (e.g. Cohen's *d*, Pearson's *r*), indicating how they were calculated

Our web collection on statistics for biologists contains articles on many of the points above.

Software and code

Policy information about availability of computer code

Data collection	No software or code was used for data collection.
Data analysis	Phylogenetic analysis was performed using MAPLE v0.3.3, BEAST v1.10.5, Tracer v1.7.1, and TreeAnnotater 1.10. Epidemiological inference was performed used EpiNow2 v1.5.2 and software generated by the REACT SARS-CoV-2 studies. Simulations were performed using julia v1.10.2 based on code from Endo et al., 2022 (Science). Post-processing was performed using python v3.10.10, R v4.0.2 and R v4.3.2. Treeswift v1.1.42 was used for tree post-processing. Baltic v0.2.2 was used for tree visualization.<br>Each of these is documented and made available through https://github.com/pekarj/nyc_mpox_phylogeography.

For manuscripts utilizing custom algorithms or software that are central to the research but not yet described in published literature, software must be made available to editors and reviewers. We strongly encourage code deposition in a community repository (e.g. GitHub). See the Nature Portfolio guidelines for submitting code & software for further information.

Data

Policy information about availability of data

All manuscripts must include a data availability statement. This statement should provide the following information, where applicable:

- Accession codes, unique identifiers, or web links for publicly available datasets
- A description of any restrictions on data availability
- For clinical datasets or third party data, please ensure that the statement adheres to our policy

MXPV genomes from the NYC DOHMH PHL that we generated have been deposited at NCBI (BioProject PRJNA949682). The accession IDs for all MPXV genomes used can be found in Supplementary Table 1 (all) and Supplementary Table 2 (GISAID). The genomes available at NCBI are public; those at GISAID require registration with GISAID. HIV surveillance activities are protected by New York State Redisclosure Law Articles 21 and 27-F, which prevents the submission of HIV-1 genetic sequences to public databases. Data were shared with investigators under data use agreements with UC San Diego. Data requests can be made by emailing ftaki@health.nyc.gov, with an expected time for a response of 1 week. Time to provide the data depends on the data request and the potential need of a data use agreement.

Research involving human participants, their data, or biological material

Policy information about studies with human participants or human data. See also policy information about sex, gender (identity/presentation), and sexual orientation and race, ethnicity and racism.

Reporting on sex and gender	Gender of individuals was available via existing public health surveillance data by DOHMH surveillance analysis. It was self-reported. Gender was included in the multivariate logistic regression model, but we do not share individual-level data.
Reporting on race, ethnicity, or other socially relevant groupings	Race/ethnicity and sexual orientation was available via existing public health surveillance data by DOHMH surveillance analysis. It was self-reported. These characteristics were included in the multivariate logistic regression model, but we do not share individual-level data.
Population characteristics	We included age, race, ethnicity, gender, and geography in the multivariate regression. We additionally had access to HIV diagnosis and MPXV vaccination status.
Recruitment	These are individuals monitored via existing public health surveillance infrastructure in NYC. Nearly all the sequenced mpox cases were from sexual health clinics, which might not be entirely representative of citywide cases, but are likely a sentinel population with which to understand sexually transmitted pathogens in NYC.
Ethics oversight	This was a routine analysis of existing public health surveillance data by DOHMH surveillance analysts and thus not subject to Institutional Review Board approval at the NYC DOHMH. The IRB of the University of California at San Diego, which does not conduct public health surveillance, judged the analysis to be minimal risk [research], and “a waiver of individual authorization for the use of Protected Health Information (PHI) was granted as stipulated by the HIPAA Privacy Rule, 45 CFR 164 section 512(I).”

Note that full information on the approval of the study protocol must also be provided in the manuscript.

Field-specific reporting

Please select the one below that is the best fit for your research. If you are not sure, read the appropriate sections before making your selection.

☒ Life sciences ☐ Behavioural & social sciences ☐ Ecological, evolutionary & environmental sciences

For a reference copy of the document with all sections, see nature.com/documents/nr-reporting-summary-flat.pdf

Life sciences study design

All studies must disclose on these points even when the disclosure is negative.

Sample size	No sample size calculations were performed. All genomic data available at the time of writing were considered, resulting in 757 high-quality MPXV genomes from NYC, 1127 from the broader United States, and 2160 from the rest of the world. Considering that a large proportion of MPXV genomes available are from NYC, we believe this is a sufficient sample size to characterize MPXV transmission in NYC.
Data exclusions	The earliest high quality genome was used per mpox case in the analysis. Any additional genomes for a given case (when known) were excluded because we are examining transmission of MPXV in NYC and including multiple virus genomes for a given individual in the inference would lead to inaccurate results.
Replication	Epidemiological inference was performed using multiple approaches to properly characterize the transmission of MPXV in NYC. Code is available so that results can be reproduced.
Randomization	Not applicable as our study was a retrospective analysis of a public health surveillance cohort and not a clinical trial or cohort study.
Blinding	Not applicable as our study was a retrospective analysis of a public health surveillance cohort and not a clinical trial or cohort study.

April 2023

Reporting for specific materials, systems and methods

We require information from authors about some types of materials, experimental systems and methods used in many studies. Here, indicate whether each material, system or method listed is relevant to your study. If you are not sure if a list item applies to your research, read the appropriate section before selecting a response.

Materials & experimental systems

n/a	Involved in the study	
☒	☐	Antibodies
☒	☐	Eukaryotic cell lines
☒	☐	Palaeontology and archaeology
☒	☐	Animals and other organisms
☒	☐	Clinical data
☒	☐	Dual use research of concern
☒	☐	Plants

Methods

n/a	Involved in the study	
☒	☐	ChIP-seq
☒	☐	Flow cytometry
☒	☐	MRI-based neuroimaging

Plants

Seed stocks	N/A
Novel plant genotypes	N/A
Authentication	N/A
